# Children’s Play Environment after a Disaster: The Great East Japan Earthquake

**DOI:** 10.3390/children2010039

**Published:** 2015-01-28

**Authors:** Isami Kinoshita, Helen Woolley

**Affiliations:** 1 Department of Landscape Architecture, Chiba University, Matsudo 271-8510, Japan; 2 Department of Landscape, The University of Sheffield, Sheffield S10 2TN, UK; E-Mail: h.woolley@sheffield.ac.uk

**Keywords:** play, disaster, post-traumatic stress disorder (PTSD), adventure playground, health triangle, safety, radiation

## Abstract

The Great East Japan Earthquake of March 11, 2011, together with the subsequent tsunami and nuclear power station accident, damaged a wide area of land. Children who experienced these terrible disasters and the post-disaster situation are still suffering in mental, physical and social ways. Children’s play is an activity that they undertake naturally and which can help them recover from such disasters. This paper addresses the role of play, adventure playgrounds and other play interventions, including play buses, for the health triangle, which addresses mental, physical and social issues of children after the disasters. These interventions were shown to be effective because children could express their stress. This included play for their mental health, different body movements for their physical health and communication with playworkers and new friends for restructuring their social health. These three aspects relate to and support each other within the health triangle. An increase in childhood obesity and lack of exercise is an additional health issue in Fukushima. For a balanced recovery within the health triangle, more play environments should be provided and some improved. A child’s right to play should be implemented in the recovery stage after a disaster.

## 1. Introduction

The World Health Organisation (WHO) describes health as a “complete state of physical, mental and social well-being (WHO, 1948 [1]) and this definition has been explained by some as a health triangle (Jenty [2], Ireland [3]). This concept of a health triangle was suggested by Araska Middle School in 1997 [4], when a graph was used to show the relation between the three different dimensions of mental, physical and social health.

Post-Traumatic Stress Disorder (PTSD), which can occur after a traumatic event, is one expression of a person not being in a complete state of good health. The phenomena of PTSD is classified into three types; (1) Re-experiencing the traumatic event; (2) Avoiding reminders of the trauma and (3) Increased anxiety and emotional arousal (Saylor, 1993 [5], Okuyama, 1996, [6]). Post-traumatic stress disorder (PTSD) suffered by children as the result of a disaster has been discussed by authors such as Saylor (1993) [5], who suggest that the symptoms differ depending on factors such as gender and age.

Although PTSD was understood to be a health issue in many parts of the world, it was not recognised in Japan at the time of the Kobe earthquake in 1995. However, the importance of play was understood by playwork volunteers from Setagaya Play Park in Tokyo who created an adventure playground in Kobe. The adventure playground was criticized, mainly by adults, because there was such a contrast between the children’s play and the sad, mourning atmosphere of people who had lost families, friends, houses and all their resources in the earthquake. At times in the adventure playground children played “earthquake play” for which the playworkers and the executive organization were strongly criticized.

After the Kobe Earthquake, PTSD was recognized in increasing numbers of patients and as a result the importance of children’s mental health care was acknowledged. As one aspect of mental care in Kobe area, pediatric doctors advocated children’s play as one aspect of mental care to help release their stress and prevent PTSD by bringing children back to increased levels of normality in their daily lives (Okuyama, 1996) [6].

The UNISDR reports that, “30–50 percent of fatalities arising from natural events are children. The main causes of mortality in children are usually the same conditions that cause morbidity in non-emergency settings—children are vulnerable but disaster risk reduction can help minimize the risks from hazards” [7]. Disaster risk reduction can be improved by the daily play activities of children which allow them to know their local environment and to develop their risk management capability. In the case of the Tohoku triple disaster, the evacuation activities of children varied depending on how well they knew their locality which had been developed and absorbed through play in their daily lives. The effectiveness of evacuation was further influenced by children’s training for such events. This is epitomized in the famous story known as known the Miracle of Kamaishi (Katada, 2012) [8], where nearly all 3000 children from the elementary and junior high schools were saved because of their repeated training in tsunami evacuation drills. It was also reported that in different areas children survived by climbing to higher ground they were familiar with in their daily play (Appendix 1) [9].

However, what happened to children’s health after the triple disaster? Drawing upon the medical experience of the Kobe earthquake, the main focus on children’s health after the Tohoku disaster was the prevention of PTSD, including flashbacks of the disasters. To this end, the care program concentrated on sending counsellors to the area. Professional counsellors were the only people working with children to help minimize their experience of flashbacks. Initially, there was no public program to provide playgrounds and promote children’s play for their health. One year after the disaster, children in temporary housing had little, if any, play space (Woolley and Kinoshita, 2014) [10]. Four important factors in support of children’s play were identified: Space, People, Interventions and Time. Adventure playgrounds, mobile play buses and cars and indoor playgrounds were initiated in the disaster area by both the voluntary and private sectors.

The United Nations General Comment No.17 states the challenges to be addressed in the realization of article 31 of the CRC (Convention on the Right of the Child). This asserts children’s right to play, the lack of recognition of the importance of play and recreation, resistance to children’s use of public spaces, balancing risk and safety, pressure for educational achievement, overly structured and programed schedules, neglect of article 31 in development programs, a lack of investment in cultural and artistic opportunities for children, the growing role of electronic media and marketing and commercialization of play [11]. As Japan was the only industrialized country of the eight consulted about article 31, it could have been expected that Japan would implement all these assertions. It appears, from an overview of post disaster policies for the whole area, that such challenges of cultural and social context may appear clearly after the disasters had happened. However, as already mentioned, there were some interventions such as adventure playgrounds, mobile play busses and indoor playgrounds which have contributed to play opportunities for children after the disaster. Here we will explore the relationship between health and those interventions.

As Masten and Narayan (2012) described, “research on pre- and post-disaster interventions to promote resilience in young people and families in mass trauma situations is a top priority [12]”. However, there is not enough scientific data on this research. Joy and Howard Osofsky researched the aftermath of the disaster Hurricane Katrina. They summarized that “to sensitively help with evacuations and return to normalcy, responders must also be trained to understand the culture and traditions of the affected communities [13]. NPO Japan Adventure Playgrounds Association has been trying to establish adventure playgrounds so that they can be sustained by the local community. Some have criticized that “Resilience-building interventions may be ineffective and perhaps even harmful [14]”. Thus, obvious interventions should be used carefully and attention paid to possible, unintentional effects. As Japanese adventure playgrounds include a strategy of community involvement, this intervention is similar to a community building strategy, as discussed in the case of Katrina’s Children (Joy and Howard Osbone, 2007) [15].

## 2. Methodology

This article will now explore how children’s play is supported in different contexts through interventions such as adventure playgrounds, play buses and cars and indoor play facilities and how these have become part of the provision for children’s healthcare between the Kobe earthquake in 1995 and the Tohoku triple disaster in 2011. The paper does not address issues of physical health in a quantitative way but focuses on qualitative aspects of mental and social health with some relationship to physical health [16]. This paper is not based on evidence from a purely medical perspective, rather it reports some aspects of how children’s play environments relate to the health triangle; It does this by taking a qualitative approach in the form of informal conversations and more structured conversations over the period between 2011 and 2014. Some of these conversations were held between the first author and play work staff and some involved both authors. The observation and interview research by both authors were conducted in spring of 2012 and 2014. The first author conducted additional interviews by telephone and e-mail. The purpose of the process was to establish the meaning of play in the reconstruction strategy, where some intervention was needed for the betterment of children’s health. This may be considered as action research involving a responsive and iterative semi-structured approach. All the people interviewed were keen for their opinions and experiences to be shared with others worldwide.

## 3. Results and Discussion

### 3.1. Play in a Post Disaster Context

There was a clear professional focus on mental health and PTSD in the post disaster area of Tohoku, which is of course important, but children also need to develop their resilience and ability to overcome stressful events. The care children might need can vary depending on their situation. For children experiencing trauma, support from a professional counsellor is important. Others with less severe symptoms can be supported in alternative ways.

Children’s play can offer benefits to all three domains of the health triangle, each of which relates to the others. A focus on social relationships might also remove children’s feelings of anxiety and encourage them to engage with other people.

Children’s play may also contribute to brain development (Schonkoff, 2000) [17]. Free play can have a very important meaning linking physical and mental health (Play England, 2009) [18], while playing and walking provide children with more physical activity than most other activities (Mackett, 2004) [19].

Children’s play has a role in releasing stress and PTSD; “Play is essential to the social, emotional, cognitive, and physical well-being” (Mulligan, 2012) [20]. Additionally, “Play and sporting activities are one of the best ways for children to deal with stress.” (Child to Child Trust, web-site) [21].

Lisul (2004) [22] explained that play was a coping strategy after a stressful event. In the case of the bombing in Yugoslavia in 1999, she reported that a special type of repetitive (traumatic) play often occurred which was compared with the usual (creative) play, at a primary school, in Novi Sad, which had been heavily damaged during the bombing. Lisul (2004) reported that, from observations of children aged 6 and 7 years old, 19 of 23 children had an active and positive attitude to the traumatic situation. Among these 19 children, 13 were playing. She described “Most of these plays were ‘war games’ full of fights and anger, but children were able to think of reasons for war and solutions that would stop it through games. In some of the play, children were observed showing some characteristics of traumatic play and they were very precise in pointing out and explaining this. This is very important, because traumatic war games increased some problems and fears, and the children’s ability to find solutions helped in their recovery” (Lisul, 2004) [22].

This understanding of traumatic play can be used as a lens to explore some of the play experiences of children in Japan in the aftermath of the earthquake and tsunami. Traumatic play such as earthquake play and tsunami play were seen in the adventure playgrounds. However, a discussion is still taking place in Japan as to whether this is a good thing or not, with some victims claiming that the atmosphere should be serious, not playful and others thinking that this might remind children of the stressful events but help to prevent flashbacks and PTSD.

### 3.2. Adventure Playgrounds and Conflict in a Post Disaster Context

There is not enough understanding of the role of adventure playgrounds after a disaster, although adventure play-based therapy for children with PTSD has been advocated [23] (Tucker, 2014).

The first adventure playground at Emdrup in Copenhagen was created during wartime in 1943, where children might play war games in the debris. Prof. Sorensen observed that children liked mischievous play and building using scrap wood found on a building site (Hurtwood, 1968) [24]. Hurtwood summarized the meaning of the adventure playground as “children build order in the chaotic situation” and introduced the concept of adventure playgrounds to England. So, the role of an adventure playground for children after a disaster, can be as Lisul mentioned “finding solutions helped in their recovery”.

Arvid Bengtson explained that an adventure playground is an essential outlet in this situation of stress (Bengtson, 1972) [25]. Children can get rid of their superfluous energy and aggression can be sublimated. An adventure playground under good supervision can provide a repression-free area where children can develop independence and learn to stand on their own feet. This is not only beneficial to their physical health but also to their psychological well-being.

Duttner (1969) [26] explains the role of play as a control of experience based on Jan Piaget’s theory that assimilation and accommodation of the local environment contribute to knowledge, understanding and development of people. Specifically, Duttner (1969) suggested that playgrounds can be similar to adventure playgrounds having opportunities for assemblage and dis-assemblage, including old tires, scrap lumber (with edges rounded), rope, cable spools, bamboo poles, canvas and burlap sacks, blocks, automobile parts, old mattresses, chairs, cardboard cartons, indeed an endless list of parts [26]. Noren-Björn wrote that the meaning of play for mental development was also based on Piaget’s theory and Lill Peller’s play theory. She further explained the role of play as part of the social and physical development of a child, from her survey at both unsupervised and supervised playgrounds (play park, adventure playground). She clearly understood that there was a greater variety of play involving social and physical development in a playground where there were playworkers [27]. The impact of play and adventure playgrounds in relation to the health triangle is demonstrated in Table 1 compared to counselling which had been provided as a government program.

**Table 1:** Health triangle and tasks for anti PTSD.

	Conditions Required to Support the Reduction of PTSD *1	Counselling *2	Play, Adventure Playground *3
Mental health	Daily task; deal with everyday issues; less stress; feeling safe; balance in lives.	In a case of serious PTSD, need to be cared for by counsellors, overcome by talking over time.	Emotional experience, If not so severe, to prevent PTSD, by overcoming through expression in play.
Physical Health	Body condition rest, exercise and nutrition; no harmful substances; no disease	Body condition (physical health) relates to mental condition.	Moving about, inspired by the situation, releases stress and promotes physical development.
Social Health	Make/keep friends; get along well in society; communication; feeling not alone; sense of “I did it” (achievement).	Trust with the counsellor, and supporting volunteers.	Playing with friends; communication with friends, playworkers and other adults in the playground; social life in the community

References: * 1 Jenty (Webpage, 2014) [2], Lisul (2004) [22], Ireland (Webpage, 2014) [3], *2 Okuyama (1965) [6], F.Saylor (1994) [5] *3 Shonkoff (2000) [17], Child to Child Trust (2014) [21], Tucker (2014) [23], Hurtwood (1968) [24], Bengtson (1972) [25], Dutner (1969) [26], Noren-Björn (1982) [27].

There is little evidence to prove the importance of play in children’s development in the context of disasters. However, there was a Japanese government research report which demonstrated evidence that children who played in nature had more sense of justice than those who did not (MEXT, 2012) [28]. Therefore, the evidence is currently only partial. However, it may be considered that play is too general a term to identify its benefits in this type of situation and that what may be important is the type of play children undertake; there is little evidence as to whether traumatic play is positive or negative. It is possible to hypothesize that it may be a mixture of both, as was seen in the adventure playgrounds in the aftermath of the disaster and as is further discussed in the rest of the paper.

### 3.3. Kobe Earthquake and Adventure Playground

The Great Hanshin Earthquake (Kobe Earthquake) occurred at 5:46 on January 17, 1995. It measured 6.8 on the moment magnitude scale and M 7.3 on the Japan Meteorological Agency (JMA) magnitude scale. Approximately, 6334 people died, three people are still missing and 43,792 people were injured. Kobe city was seriously damaged. This was the worst earthquake disaster in Japan since the Great Kanto Earthquake of 1923.

Because many volunteers came to help and support the disaster area in activities such as rescue, evacuation centre activities, building temporary houses, repairing damaged houses and reconstruction, “volunteer” and “Non-Profit Organization” became popular keywords in Japan and resulted in the establishment of the Non-Profit Organization (NPO) Act in 1998.

Hideaki Amano was the first playworker at Hanegi Play Park in Tokyo (Appendix 2) and he went to Kobe on January 25, 10 days after the catastrophe, as a representative volunteer from Setagaya Play-Park. He and the other playworkers from Setagaya started an adventure playground activity at the corner of the park in the Nagata ward. At that time, about 250 people were sheltering in the park. Amano and his colleagues of Setagaya Play Park worked at this adventure playground for 5 months. At that time, PTSD was not recognized and the impact of children’s play was not understood by the people around the playground, rather it offended them. Indeed, the adults around the playground became angry, when the children played earthquake play. It is easy to understand how the adults became angry in such a situation because most people were mourning the loss of family members or friends, houses, other property and social networks. The contrast between these lost physical and social landscapes and the children’s play landscape in the adventure playground might have been offensive to some of the adult victims.

However, children are different from adults. They do not have property. Even though they had lost family members and friends and experienced a great shock, they would play if others were playing. Gradually they could express their inner trauma through play over time. As Amano has stated, not only the consultants involved in the mental care of children, but also specific types of play environments were needed in the evacuation and recovery process after the disaster.

In a similar way that Lisul described in Yugoslavia, traumatic play was also seen here in Japan. At first, there were children who were irritated, exploring anger and being extremely aggressive. This was symptomatic of PTSD, type 3: increased anxiety and emotional arousal. The children who had shown the symptom type 2: avoiding reminders of the trauma, which is probably considered to be not as serious, joined in play influenced by the other children. The symptom of type 1: re-experiencing the traumatic event was evidenced in earthquake play. At that time, Amano and the other playworkers, together with other adults around the playground, were not aware of PTSD.

Amano explained traumatic play and the impact of the adventure playground in children’s recovery in the aftermath of the Tohoku disaster in a newspaper article. He describes the transformation of traumatic play to creative play over time:

*“It was the amusement place of the child” where I placed myself by force with a blue sheet at one corner of the refugee tent area in a park.*

*At every distribution of boiled rice, a queue of almost 100 meters is made. We played with a child while roaring with laughter just beside the line. Both the sufferers of the disaster and the volunteers looked dubiously at the scene of our playground while emergency relief was still needed. Victims gathered day by day, and “the amusement place of the child” was frowned on.*

*However, everywhere was a refugee tent village, and both the school grounds and the park were full, and the place where a child could play freely did not exist anywhere.*

*Adults came to talk about their experiences of suffering in the month after the disaster. I hoped that a day would come when a child would express such an experience. After two months it was expressed in play.*

*“Seismic intensity 1 2 3!” 6–7 children get on the handmade desk which they had made with plywood and they huddle and rise slowly. “Seismic intensity 6 7!” The leg of the desk, where the children are, breaks and so they fall down flop. Ah! The shout of joy of the children rings through the air.*

*Children show scraps of wood and they scrunch up newspaper and place it in a bin. They set fire to the end of the paper and flames rise while they look up. “They are starting to burn! A town burns, a school burns! They pile up more scraps of wood while crying and create a roaring fire. “Ooh!” a cry of joy.*

*For the adults who suffered from the disaster, this scene was unpleasant. However, it was one month before even an adult came to talk. The child who does not have words to talk about what is in his heart only has play as a way of expressing themselves. “Seismic intensity 7!” they break the leg of the desk, and your mind tries to understand the huge, unreasonable experience that they can never accept that it is easier to “burn” a town than it is to build it again.*

*This play might result in a child getting over and accepting the event which had happened.*

*Play is not simply about killing time. The children who suffered from the disaster must get over it by themselves somehow. They run around and blow off stream. They have a long talk with friends. They change an event that sometimes involves a serious injury into play that they can accept. The children build their world and express themself and heal themself.*

In an unprecedented disaster, a child is obviously a victim. It is good to let a counsellor enter the stricken area to care for their emotional well-being, but a child has power to heal themself. Therefore there should be enough places for play”.

[29]

(From part of the article written by Hideaki Amano, Power of Play Healing Children, the Kyodo News, March 14, 2011. Translated by the authors.).

### 3.4. The Great East Japan Earthquake and Adventure Playground

The Great East Japan Earthquake (Tohoku Disaster happened at 14:46 on March 11, 2011. Its moment magnitude was M. 9.0 which was the biggest one in the history of Japan. Most of the coastal area was damaged by the Tsunami. The height of the tsunami was more than 10 m and 40.1 m at its highest, depending on the geographic condition. By June 14, 2014, 18,502 people had lost their lives or were missing. More than 400,000 houses and buildings were partially or completely destroyed. The number of evacuees peaked at more than 400,000 people but by May 15, 2014 [30] this had reduced to 258,219. The affected area was immense and included the coastal area of municipalities from north of Iwate prefecture to south of Chiba Prefecture, a distance of more than 500 km.

The nuclear power station accident caused by the earthquake and tsunami, which resulted in a meltdown of three of the plant’s six nuclear reactors, increased the influence and seriousness of the damage. This incident measured Level 7 on the International Nuclear Event Scale, which is the same level as that of the largest nuclear incident in the world: the Chernobyl Disaster in 1986 [24]. Because of radioactive contamination, people had to evacuate immediately although their houses were and still are undamaged by the earthquake and tsunami. The incident resulted in the evacuation of over a further 300,000.

This triple disaster is unique in the recorded history of the world. Although it is now more than three years on, many people are still living in temporary houses and many young families have decided not to go back to their home town and the population of the municipalities which suffered from the disaster has been decreasing, with the younger generation particularly affected.

The NPO Japan Adventure Playgrounds Association (JAPA) started a project building an adventure playground in the disaster area after, learning from the experience of the Kobe Earthquake and recognizing the importance of supporting play in the recovery of children suffering from the disaster. JAPA looked across a wide area for a suitable place to build an adventure playground and found a place at Ohya district on a hillside behind Ohya primary school where people had evacuated from the lower coastal area in Kesennuma city.

Preparatory work started in April 2011 and the playworker from Hanegi Play Park was sent to this new location. His nickname was “Kanpei”. Hideaki Amano and other playworkers came to help build the playground but children who had been evacuated to the school also helped. It was quite difficult to distinguish whether they were working or playing. In this case, Hideaki Amano and the other playworkers developed communication processes to get agreement to build the playground with the people of the community, the people who had evacuated at the school and teachers of the school. Through this process, two land owners kindly offered the use of their hillside land for the adventure playground because the land was not being used. Although adults had heard about this playground, they did not understand it. Some said “there are many natural spaces around this mountain for children, why do we need to make such a playground?” However, when they watched children gradually recover their smile by working and playing while building the playground, the adults also recovered their smile. Someone said “what a wonderful thing it is, to be able to see the children’s smiles”.

**Figure 1:**
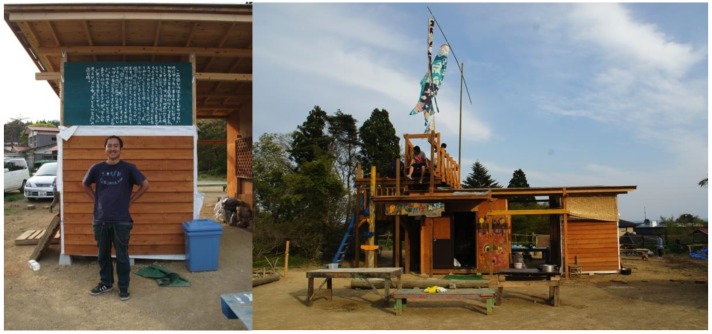
Adventure playground Asobi-ba in Kesenuma, playworker Kanpei (left).

This adventure playground was named “Asobi-ba” (Figure 1), which in Japanese means playground. It has been gradually recognized and supported by the local community. Kanpei played a great role in communicating with local people to get their trust. An event involving the local community and residents of the temporary houses in the school ground, demonstrated the very good and close relationship between adults and children. In such a way, the health triangle of mental, physical and social domains looked very positive and different from other temporary housing sites.

Asobi-ba was mentioned in the mass media such as newspapers and television because it was attractive for the media to highlight what was going on in the disaster area. Media coverage increased the number of visitors to Asobi-ba. Among the visitors were those who were very impressed and wanted to build this type of adventure playground in their neighbourhood. After the initial success of the Ohya playground, adventure playgrounds and playground activities increased across the Tohoku area to about 25 as shown in Table 2.

Another playworker, nicknamed Buncha, came to support the adventure playground movement in other areas. In Koizumi district, again in Ishinomaki city, he helped with the building of another adventure playground. He also supported other areas if people wanted to build an adventure playground by consulting and helping during the initial building period. He also helped by training young playworkers. He responded to the question asking if there was tsunami play in children’s play.

*“Tsunami play was seen often at Asobi-ba. With a handmade equipment slide, a child gliding from the top plays the part of the tsunami. A child standing below is drenched, and may die or not, and at the side another child is positioned in the role of announcing “a major tsunami warning is announced”. They all survived on this occasion. Hideaki Amano (who sometimes came from Tokyo to support us as the headquarters of the adventure playgrounds network JAPA), said that the big slide had been made with tsunami play in mind. In the summer time, children played by flushing water, brought from a keg, from the top of the slide, as tsunami play. There was another child who was frightened feeling the reality of the Tsunami by watching this play. Then I told the children to stop this tsunami play. We (the playworkers at Asobi-ba) have never made the kids play tsunami play. However, another adventure playground organization said that they had tried to involve children in tsunami play. They had tried to use a blue sheet and balls to imitate a tsunami. At Asobi-ba, we let children decide by themselves whether to play tsunami play or not. There is a discussion whether it is good or not for play-workers to introduce this kind of tsunami play for children so that they can release their stress and trauma.”*

**Table 2:** Adventure playgrounds in the Tohoku Area. (original data was given from Mr. Tsutom Sunaga to use with his permission. This shows the situation on July, 2012 which movement is growing.)

Address			№	Name	Management				Organization Type
Prefecture	Municipality	District			①	②	③	Opening Frequency	
Iwate	Noda vilage		1	Noda Primary School Playpark	○	-	Ⅰ	irregularly	C
	Rikuzentakata	Kurosaki	2	Makibakko	○	♪	-	-	B + D
Miyagi	Kesennuma	Motoyoshi	3	Asobiba	○	♪	Ⅳ	5days /week	E→B
		Motoyoshi	4	Asobo-car	○	♪	Ⅳ	irregularly	E
		Motoyoshi	5	Kurinoki Hiroba	○	♪	Ⅳ	4/week + holidays	B
	MinamiSanriku town	Utatsu	6	Utatsu TengunoYama School	○	-	Ⅲ	2/week	A
	Tome city		7	Tome no Asobiba	○	♪	Ⅰ	irregularly	A+E
	Kurihara city	Kannari	8	HieJinja no Asobiba	○	♪	Ⅰ	irregularly	A+E
	Ishinomaki city	Kitakami	9	Nikkori Playpark	○	♪	Ⅰ	irregularly	A
		Kawakita	10	Kamegamori Adventure Playground	○	♪	Ⅱ	1stSunday/monthly	B
		Ishinomaki	11	Playpark Yappesu	○	♪	Ⅰ	1/monthly	C
		Ishinomaki	12	Koganehama Chibikko Hiroba	○	♪	Ⅲ	every weekend	D
		Ishinomaki	13	Ishinomaki Playpark in Kaihoku school	○	♪	Ⅰ	4th Sunday	C
		Ishinomaki	14	Ishinomaki Playpark in Sumiyoshi	○	♪	Ⅰ	Second Sunday	
		Ishinomaki	15	Minatoprimary schoool Asobiba	○	-	Ⅰ	irregularly	A
		Ishinomaki	16	Nikoniko Playpark	○	-	Ⅰ	1/monthly	D
	Rifu town		17	Rifu Playpark	◎	♪	Ⅰ	irregularly	A
	Sendai city	Aoba ku	18	NishiKoenPlaypark	◎	♪	Ⅳ	3 or 4 times a week	A
		Wkabayashi ku	19	Furujiro playpark	◎	♪	Ⅱ	1/monthly	A
		Wkabayashi ku	20	KaiganKoen Adventure playgrounda	●	♪	Ⅰ	closed by disaster	
		Wkabayashi ku	21	Rokugo Asobiba	○	♪	Ⅲ	1/weekly	C+E
		Wkabayashi ku	22	Nipperia Asobiba	○	♪	Ⅱ	1/weekly	
		Wkabayashi ku	23	Shichigo Asobiba	○	♪	Ⅱ	1/weekly	
		Wkabayashi ku	24	Arai2goKoen Asobiba	○	♪	Ⅱ	1/weekly	
		Wkabayashi ku	25	KamiaraiKokaido Chibaihiro	○	♪	Ⅰ	1/weekly	
		Wkabayashi ku	26	Playgroup at temporary houses at the site for school	○	-	Ⅲ	1/weekly	D
Yamagata	Yamagata city		27	Yamagata Playpark	○	♪	-	irregularly	A
Fukushima	Minamisoma city		28	Minna Republic	○	♪	-	Irregularly	C
	Date city		29	planning	☆	-	-	-	A
	Fukushima city	Iizaka	30	Moniwa Adventure playground	○	-	-	2-4 days/week	A
Sources: NPO Japan Adventure Playgrounds Association									
[index ①: starting time] ◎：from before the disaster ●closed by disaster ○：started after the disaster ☆：planning									
[index ②: visiting play car 1・car 2] ♪：visiting [index ③：opening times (at moment July 1, 2012) ]Ⅰ: less than 10、Ⅱ: 10～49、Ⅲ: 50～99、Ⅳ: over 100									
[index organization type]: A: local group, B: Neighbourhood Community, C: Network of Local organizations, D: Volunteer Organization from Outside, E: Network organization in Japan, F: Schools/Universities									

An earthquake disaster might make children violent just after it had happened. But it was observed that these children at the playground gradually recovered their tenderness again over time.

When the kid’s hideout play got popular among the children at Asoi-ba, they always took items such as water, food, radio and flashlight or torch. They would say: “Tsunami may come again. In that case, we evacuate to here. Here is surely a safe place.”

This highlights traumatic play and its role in helping children overcome trauma and in preventing flashbacks. The interview with Buncha and Kanpei demonstrates that playworkers know the children who frequently used the adventure playgrounds and their different backgrounds. Children changed through communication with the playworkers and other children.

Following the Kobe earthquake, Okuyama (1996) [6] described that children’s play relating to their frightening experience or slight regression was a behaviour to protect them from their uneasiness. Then, she concluded that it was very important to create an environment in which children felt safe and protected by the people close to them. For that, the adults surrounding the children should be stable. If they were not and were uneasy, or sometimes angry, resulting in conflict or fighting, then children’s mental condition could deteriorate. For that reason, she argued the importance of supporting both children and their parents to help release their stress and uneasiness. For children themselves, she advocated the importance of a playground, so that they could deal with their stress and uneasiness by themselves. She analyzed that children expressed feelings of uneasiness and their anger through the process of play. She stated that sometimes in their play the symptoms of PTSD, such as: re-experiencing the traumatic event, avoiding reminders of the trauma and increased anxiety and emotional arousal were sometimes exhibited. However, communication and sharing experience with friends would help to calm the situation in the knowledge that other children had experienced and felt similar things. From her research after the Kobe disaster, she realized that it was very important to open a playground to help children recover their emotional balance, as well as enabling them to restart school and preschool sooner. This was similar to the experience described by Hideaki Amano. In the case of Asobi-ba in Kesennuma after the Tohoku disaster, Kanpei had explained that listening to children and their communication with playworkers had also been important and in their care they gradually opened up and talked. This enabled him to gradually get to know their family. In turn, those families who came to learn of their children’s play through communicating with playworkers, also recovered their calm voice and attitude. However, some children, especially those visiting the playground every day, exhibited signs of stress at home. In this way, the role of the playworker could have been extended to that of a social worker. So, it looked as if the role of the playworker may extend to being a social worker organizing social health. Where adventure playgrounds had been supported by the local community, communication with them also had a positive impact on the children where they felt protected and able to recover their emotional balance.

When we asked Buncha if there had been an improvement in the understanding of children’s play through the Asobi-ba and other adventure playground activities, he responded:

*“Here, people think, of course, it is ordinary for children to play but they also think now that children cannot win in the competition of education if they are doing ordinary things. (As UN CRC committee commented that children did not have enough time to play as a result of the competitive educational system in Japan.) In the Kesennuma area there was a traditional approach to childcare which was called Yama (Forest) Gakko (School) and which involved teaching children through playing in the forest. However, this traditional method of education had been disappearing, because increasingly it was thought that it did not contribute to success in educational competitiveness. There was no thought of educational competition after the earthquake and tsunami disasters, and therefore children could have the opportunity to play with energy. This was the situation in Asobi-ba and was very much appreciated by the people. Adults had not had enough time to think about children’s needs.”*

Asobi-ba was established by the management of the NPO Japanese Adventure Playground Association (JAPA) which meant it was initiated from outside the local community. However, the management of this playground was undertaken by the local community from the summer of 2012.

One specific characteristic of Japanese adventure playgrounds is that the playground is sustained by the local community. As already mentioned, Lady Hurtwood described that the main dissenting opinion to an adventure playground concerned the perceived disorder and in some countries this is hidden from the outside by a fence. However, in Japan there is no fence around an adventure playground: it is visibly open and also invisibly open to everyone. As a common finding among about 400 adventure playground, this characteristic is often seen as a specific feature in Japanese adventure playgrounds. In Table 2, there are many cases of adventure playgrounds sustained by the local community, which have been supported by or linked with an organization from outside and these are indicated in organisation types A + E or all organisation types E.

**Figure 2:**
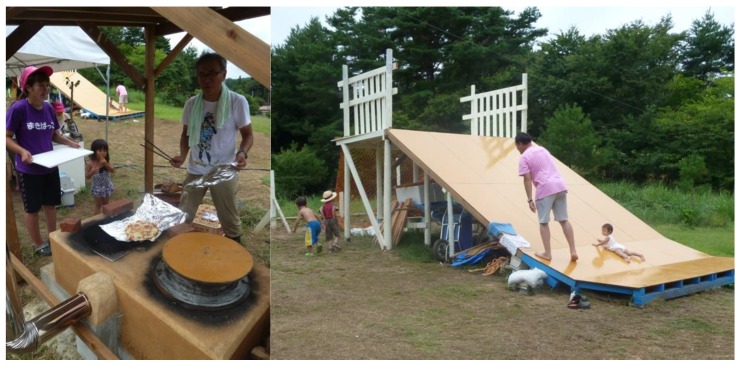
Adventure playground built by the village community “Makibakko”.

As a new adventure playground, Makibakko at Rikuzentakata is a good example to show how the local community sustained the playground. This playground was opened in the summer of 2012 and was initiated by a mother who had visited Asobi-ba. She was a member of a child rearing group at Rikuzentakata city, and she wanted to have an adventure playground at the centre of the city. However, the town centre was totally destroyed by the tsunami and the ground level had sunk by about 1 m as a result of the earthquake. The hill side for housing was limited and therefore she found some land that had been abandoned for cultivation in her parents’ village. At first she was worried that the area was very rural and that there were only four children in the village. The first author gave advice to her that the adventure playground should not have the same landscape as Asobi-ba. She could make an adventure playground in the local style of farming, inviting her colleagues in the child rearing group from the whole city. She was encouraged by this advice and opened the playground. Another key person Mr. Koizumi was also involved in this and he travelled from Tokyo making a personal sacrifice to help. Perhaps, it was remarkable that the older people of this community were delighted with this project and they took part in the process of building play equipment (Figure 2), a cooking kiln, and making a name board. It looked as though the playground was for three generations. Such a positive social situation contributes to the health triangle.

### 3.5. Play Car—Mobile Play

Sendai is a major capital city in northern Japan and has three adventure playgrounds. Kaigannkoen Adventure Playground was the largest of these but had suffered badly from the tsunami. The playworker there, Akio Nemoto, survived the tsunami by climbing to the top of the play equipment, which, in turn, stood on top of a mound. Together, with some village people who had evacuated to this playground, they survived. In this coastal area of Sendai city, the surrounding land is flat; therefore, the mound of 15 m was the highest point and indeed the only elevated piece of land in this area. As the tsunami approached, Akio wanted to be the last to leave after he had made other people evacuate from the playground. However, some old people arrived there after being evacuated from their village and climbed to the top of the mound to escape the tsunami. Akio helped the old people get warm from the cold air and snow by covering them with blue sheets and other material with which the adventure playground was equipped. Finally, they were rescued by helicopter after the mound had been surrounded by water. This demonstrated that the adventure playground might serve as a base for evacuation because survival play is familiar to an adventure playground and the relevant materials are likely to be available.

This experience was shocking for Akio, but he started play work activity by visiting the evacuation locations after just a few days of rest. The NPO Japanese Adventure Playground Association coordinated support to send play cars which were donated by private companies. One of two was used in Sendai while the other was used by Buncha to visit different sites to provide adventure playground activities supporting other activities initiated by local people.

The area of the disaster was widespread and there were enormous numbers of temporary housing sites: 260,000 people were still living in temporary houses three years after the disaster. The requirements for children’s play were not considered in the environment of these sites. Around the pre-fabricated houses, asphalt or gravel covers the surface and is occupied by many car parking places. Some sites have a volunteer play program but most sites hold these activities inside in a meeting room, not outside. It is proposed that in such circumstances, four key factors affecting play—space, people, intervention and time—should be considered for the betterment of children’s environments (Woolley and Kinoshita, 2014) [10].

**Figure 3:**
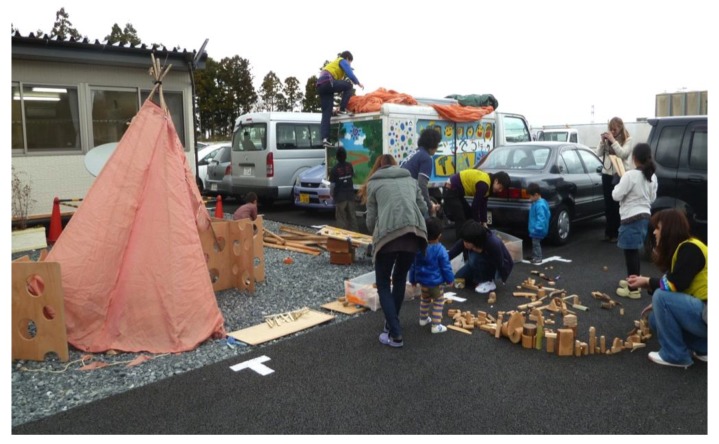
Mobile play truck at a temporary housing site.

One of the interventions to support children’s play in the temporary housing areas is play buses. Kirby provides mobile play facilities from a play bus and he has been visiting the disaster area from Tokyo two or three times every month since the disaster. He has mainly visited the area in Ishinomaki city. Ishinomaki city has a population of about 150,000 and is a local core city in the coastal area in the northern part of Miyagi prefecture. In Ishinomaki, Mr. Shibata had started play activities, using the name Rainbow Crayon, from the evacuation stage just after the earthquake and after temporary housing had been provided. He has been visiting these sites every day to support play activities for children. However, there are about 150 temporary housing sites, and it is difficult to cover the entire area. Kirby has been supporting his activities by brining play materials and playworkers to sites by bus (Figure 3). At first, their activity took place around the temporary houses using the tarmac spaces in between car parking places in front of gathering places. In some locations, it was difficult for the residents of temporary houses to understand the importance of this activity. The residents wanted to stay quiet and claimed that sometimes children made a noise. The playworkers sought to develop the residents’ understanding and gradually the residents changed their attitude to support their activity by watching children playing. Now, not only children from the temporary housing site but also children from the surrounding area come when mobile play is happening. Here, the playworker fulfils a role similar to a community worker by connecting adults with children.

There is an episode reported by Kirby about 6 months after the disaster when the play car visited a site in Ishinomaki city. A 4 year old girl came to the play car with her mother tied to her hand. When she saw other children were playing and having fun, gradually this little girl became involved and concentrated on playing with other children. Her mother was very surprised by this because this girl had not gone further than 5 metres from her side; she was always touching or following her mother closely after the disaster, which might be understood as a symptom of PTSD type 2: regression. This episode demonstrates how the play supported by the play car contributed to recovering the child’s health.

Social health for children can include not only being with each other but also being with adults. The playworker Kirby has established contact with citizen movement networks of the young generation which might be a new way of creating community and incubating activities such as social businesses. Through these contacts, Kirby has also collaborated with other organizations running the Koganehama adventure playground. He has coordinated a street party project and mini city project as big events for children to gather in the town centre. Kirby’s activity shows that the role of playworker and mobile play can connect different stakeholders to support a child’s right to play and their play environments.

### 3.6. Fukushima and Indoor Playground

**Figure 4:**
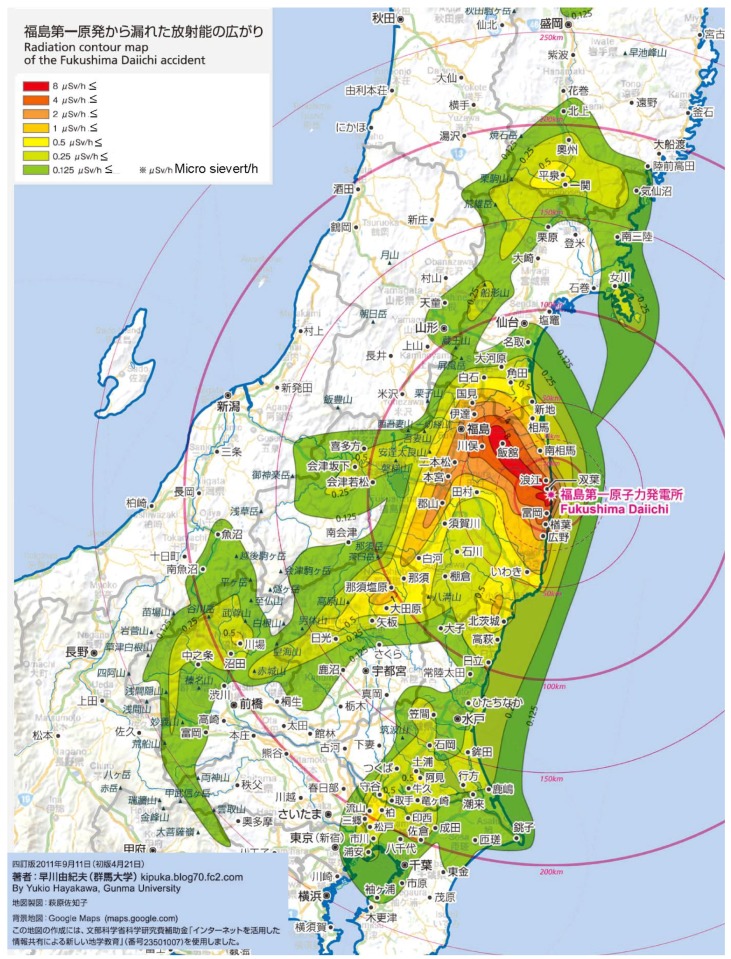
Radiation contour map of the Fukushima Daiichi Accident. This is arranged from the map made by Yukio Hayakawa with his permission (2012.3.15).

A more serious and ongoing concern is children’s environments in the Fukushima Prefecture. The area suffered radioactive contamination which spread from the power plant to the northwest (Figure 4). Young families were evacuated from the prefecture and the population suddenly declined after the disaster.

At the time, statements from the government about radiation levels were confusing for parents and resulted in fear and concern for the future of their children.

The data about radiation levels which the government and media reported for a while after the accident was measured 7 m from the ground level, well above the height of adults. Then, it changed to radiation measurements at 1 m above ground level. However, this is a standard which relates to the height of an adult. A small child’s body is nearer to the ground than 1 m where the radiation is higher and therefore children were not considered separately.

Even at a low dose, many were uncertain as to whether children should be allowed or forbidden from outdoor activities. Some parents adopted the defensive measure of restricting their children’s outdoor activities to reduce their exposure to radioactivity. However, when outdoor activity is restricted, the development of a child’s mind and body may be affected [31]. Adults worry about health impairments caused by radioactivity and children’s growth, growth because of the control on outdoor activities. The parents of small children in Fukushima continue to be faced with this great dilemma.

**Figure 5:**
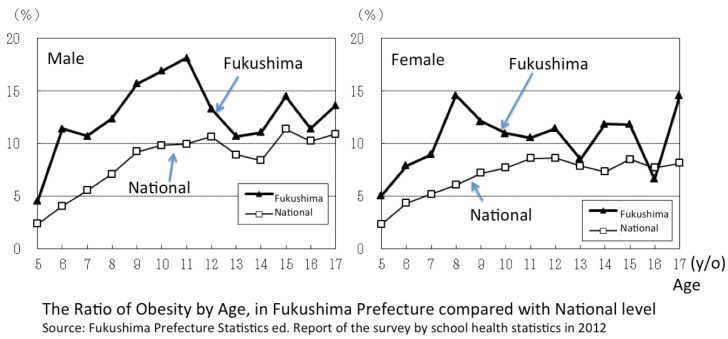
The difference of the ratio of obesity by age in Fukushima prefecture. (This is arranged from the original graph made by the department of statistics of Fukushima prefecture with the permission to use.)

The obesity of children increased noticeably in Fukushima Prefecture after the disaster (Figure 5). The ratio of children whose weight was 20% more than the standard was higher than the national average [32]. Fukushima was the top highest for obesity for 6, 7, 10, 13, 15, and 17 year old children. Also, Miyagi and Iwate prefectures, both affected by the earthquake and tsunami, were located in the higher classes of obesity. This demonstrates that children in the disaster area have increased body weights and this may be because of a lack of physical activity since the disaster. This is particularly the situation in Fukushima prefecture where there has been a restriction on being outdoors because of radiation contamination and where children’s obesity has increased since the disaster. However, obesity and trauma might have no relationship, because the numbers of children who suffered from the tsunami were limited in Fukushima to the coastal area, and most children inland were visibly not harmed. 

In Fukushima Prefecture, there has been a restriction on outdoor activity. In 2012, there were 132 public schools with this restriction reducing to 56 schools in 2013 (Nikkei News, 2013) [33]. However, there are still many parents who restrict their children from playing outdoors.

**Figure 6:**
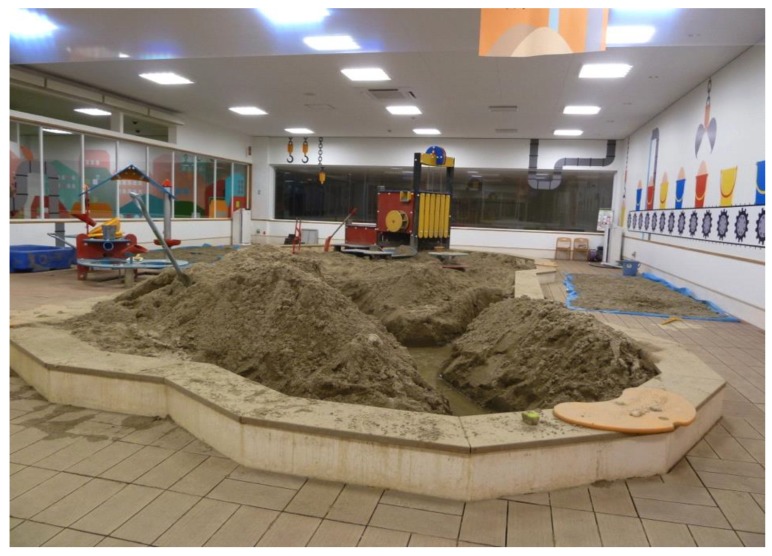
Indoor playground “PEP Kids” in Kooriyama city.

Paediatric Doctor, Shintaro Kikuchi, opened a temporary indoor playground in the summer holidays in 2011. Initially, he had tried talking to local government and private companies but with limited success. Finally, the local supermarket offered the use of a supermarket building which was closed and the city of Korriyama promised to give support with a financial subsidy. In this way, the first indoor playground “PEP Kids” opened on December 23, 2011 (Figure 6). In this indoor space, there is a sand play area of 70 m^2^ an activity zone for running, play equipment for physical movement and a cooking room. About 1000–1600 children use this daily. There are 6–8 staff in this indoor playground supervising and supporting children’s play.

**Figure 7:**
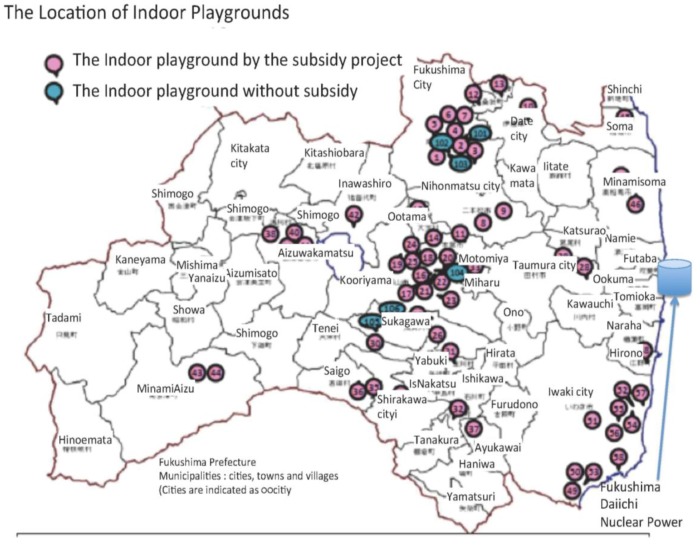
The location of Indoor Playgrounds in Fukushima Prefecture (source: Fukushima Prefecture 2014.4.7, This is arranged from the original map made by the department of Child Rearing Support of Fukushima prefecture with the permission to use.).

Then, Fukushima prefecture set up a project to support local governments to develop indoor playgrounds from 2012. The number increased to 64 indoor playgrounds in the Fukushima Prefecture by May 2014 (Figure 7). The facilities are mostly equipped by the agent handling Scandinavian play equipment. Therefore, each playground is not as different from each other as they might be. Staff provide some supervision but mostly they are not trained playworkers.

Mr. Hiroyuki Yoshino, concerned about the issue of radioactive influence on children, organized the Children and Fukushima Network. He and his colleagues in the network have been sending children from Fukushima to safer, greener places outside of Fukushima. This network extended to connect with Japanese citizen organizations supporting children at Chernobyl which developed after the Chernobyl disaster of April 26, 1986.

There was no clear information about which level of radiation was safe for children when the Fukushima Daiichi Nuclear Power Station accident happened in 2011. Initially, the government announced 20 mSv/year as the maximum safe standard level but this was criticized strongly by the public, and the government responded by changing the level to 1 mSv/year (MEXT, 2011) [34]. This means that the aerial dose rate of 0.23 μSv/h over 8 h every day became the standard for outdoors. There were many places in Fukushima, where the dose rate in the air was over 0.23 μSv/h. For the standard level to be 20 mSv/year, the air dose rate would be 3.8 μSv/h over 8 h every day outdoors. This standard was applied to decontamination workers and in deciding zoning categories for the areas where people would be allowed to live. This demonstrates that a double standard and there has been no agreement on safe levels of radiation. A young boy of 14 years old who was evacuated from Namie town, close to the nuclear power station, commented:

*“Even the scientists said different things about which level is safe, depending on their viewpoint, for or against nuclear power stations. Please come here, scientists from both sides to have a battle or discussion in front of us, then we will decide it by ourselves.” (Children’s Summit Sendai, 2011) (Appendix 3)* [35]* is might be the real opinion of children and parents who have children in the contaminated area . There are many different opinions from scientists about the impact of lower radiation levels on children’s bodies. The general public has not been able to assess meaningfully the risks and as a result has lost trust in the scientists (Appendix 4).*

[36]

Therefore, contacts with the citizen groups and organizations in Chernobyl who had been supporting children enabled Yoshino and other colleagues to identify the importance of a “sanatorium” where children could stay in a safe, uncontaminated and green environment, allowing their natural metabolism to normalize [37,38,39]. As a result, they started a program, using their network of organizations, to send children temporarily to such locations.

In Fukushima Prefecture, decontamination work has been ongoing since the disaster and, now, in most municipalities, radiation levels in the air are much reduced. The prefecture has established a strategy to support children’s play outdoors, recognizing that they cannot solve the issue of the increasing obesity only by providing indoor playgrounds. However, there are still many parents who restrict children’s outdoor play. The government has decontaminated many residential areas but, over time, higher levels of caesium have accumulated in several locations. Though the government has guaranteed the level of radiation that is safe by their measurements, there may be locations where higher levels of caesium exist and where children should be protected from its effects. Yoshino’s group and other organizations are focusing on measuring the radiation outdoors in residential areas to show more detailed data on maps so that children and their parents will be able to know where the “hot spots” are located.

In consideration of the physical domain of the health triangle in the context of Fukushima, there may be a dilemma between the impact of radiation and the promotion of outdoor play against increased obesity. However, addressing the social domain, the network of citizen and local government organizations, and professionals, including scientists with reliable data, should be encouraged to create children’s safer and healthier play environments. Fukushima Prefecture has started now to promote the adventure playground as a model of collaboration with local organizations.

It is now possible to establish the impact of the adventure playgrounds and other interventions implemented since the disaster, based on the research and discussions, using the model of the health triangle as can be seen in Table 3.

**Table 3:** Impact of interventions such as adventure playgrounds in children’s play in a post disaster context in Japan.

	Adventure Playgrounds and Other Interventions Promoting Children’s Play in Post Disaster Context
Mental Health	Expression of emotions through play: feeling accepted; communication with friends or playworkers to release stress and the feeling of uneasiness; body movement to release stress and to make positive influences to mental condition [40].
Physical Health	Moving about; different types of play result in whole body movement to promote physical development [41].
Social Health	Making friends and developing social skills; Sharing experiences with others in the same peer group; Improved well-being resulting from safe play space such as adventure playgrounds or indoor play spaces in Fukushima; Improved social interaction within a community involving not only children but also their parents and other residents.

## 4. Conclusions

It is now increasingly recognized that the mental health of children in a post disaster context may involve Post-Traumatic Stress Disorder (PTSD). However, its relationship with physical and social health should be considered. Children’s play has a very important impact on the health triangle. The CRC general comment No.17 on Article 31 stated that, there are many barriers to supporting play in Japan. This prevailing culture of not supporting children’s play appeared clearly in the post disaster context, for example at the evacuation centre and at temporary housing sites where children were restricted from playing freely.

It has been demonstrated that adventure playgrounds have an important role in helping children to release mental stress through their play. However, this needs further research to explore this more. In this research, earthquake play and tsunami play were often seen in the adventure playgrounds. Such play might be an expression of children overcoming shocking events of which, on their own, they did not understand the meaning. This is a hypothesis which merits further investigation. Through play, children might recover both physical health and social health with friends. So, the image of children recovering their smile by playing as they used to before the disaster has helped adults to understand the meaning of play. From such experience and sympathy, there are people who have been encouraged to build other adventure playgrounds in their own residential area. As a result, adventure playgrounds have developed in the disaster area. The involvement of local communities has been a feature in sustaining these developments. Further discussion about the use and integration of the health triangle may help communities develop effective health strategies.

Nevertheless, the increase in children’s obesity after the disaster in the Fukushima Prefecture demonstrates that the area may not be healthy for children. Most did not understand the importance of play and this was expressed in the restrictions on children’s play at the evacuation stage and, later, in the lack of outdoor play spaces at the temporary housing sites. Fukushima Prefecture has promoted indoor playgrounds and this might be considered appropriate at a time of radiation contamination. After de-contamination, the development of children’s outdoor adventure playgrounds, mobile play supported by interventions such as play buses and play cars and various other approaches for increasing the opportunity for children to play should be given more consideration than they have been.

## Appendix

### 1. Children that Survived the Tsunami

In the case of Togura Primary School and Secondary School in Minami Sanriku Town, children were evacuated from the public evacuation place to climb up the hills and survived. (From the research done in the April 2011 by the author). On the contrary, in the case of Ookawa primary school in Ishinomaki, while 34 children died, tragically 74 children died, because of the teachers’ error of judgment. Though, some children climbed up the mountain themselves and those who were able to do so survived.

### 2. Adventure Playground Movement in Japan

Adventure playgrounds first started in 1943 in Emdrup in Denmark, with landscape architect C.TH. Sørensen being their lead developer. This type of adventure playground was subsequently introduced by Mr. and Mrs. Ohmura in Japan, through the book “Planning for Play” written by Lady Allen of Hurtwood and translated and published in Japanese in 1973. After several experiments opening adventure playground activities, in the International Year of the Child in 1979, Setagaya ward decided on a memorial project, to build a permanent adventure playground in one corner of Hanegi District Park. This type of adventure playground was called a Play Park and was extended to four parks in Setagaya ward and then eventually all over Japan. Nowadays, there are about 400 organizations across Japan who are running adventure playgrounds including the non-permanent style, such as activities on every weekend for instance (IPA Japan, 2013) [41]. For networking purposes, the Japan Adventure Playgrounds Association was established in 2003 and supports the play activities in the disaster area.

### 3. Children and Youth Forum at the Disaster Area = Kodomo Mirai Jin Summit

At the Kodomo Miraijin Summit held on September 4, 2011, where Isami Kinoshita attended and talked with the 55 children gathered from the disaster area. This Summit was held by the support of Deutsches Kinderhilfswerk e.V. and its report was available also in English [34].

### 4. Discussion about the Safer Level of Radiation Affection

Currently, the linear no-threshold model has been used but there are still many discussions. As the reason for the safer level is not clear, it is said by many scientists and governmental officers that there has not been enough evidence or epidemiologic data about the impacts to the human body from lower radiation. In Japan, with the historical events of Hiroshima and Nagasaki, some data has been produced from epidemiologic long term research conducted by US and Japan, which was called ABCC. However, a major setback has been that the data is not open so that the others can check the safety levels [35].
